# Artificial intelligence for diagnosis and triage in oral cancer: a clinician‑centered narrative review

**DOI:** 10.1007/s10147-026-03002-5

**Published:** 2026-03-12

**Authors:** Shin-ichiro Hiraoka, Kohei Kawamura, Ryo Akiyama, Yutaka Itakura, Susumu Tanaka, Narikazu Uzawa

**Affiliations:** 1 Department of Oral and Maxillofacial Surgery, Graduate School of Dentistry, The University of Osaka, Osaka, Japan; 2 Department of Oral & Maxillofacial Oncology and Surgery, Graduate School of Dentistry, The University of Osaka, Osaka, Japan

**Keywords:** Oral squamous cell carcinoma, Artificial intelligence, Deep learning, Radiomics, Digital pathology, Lymph node metastasis

## Abstract

**Background:**

Early diagnosis of oral squamous cell carcinoma (OSCC) remains challenging, with survival largely stage-dependent at presentation. Artificial intelligence (AI) promises to enhance detection and clinical decision-making across clinical photographs, radiology, optical imaging, and digital pathology.

**Methods:**

This narrative review synthesizes peer-reviewed PubMed-indexed English-language studies up to October 2025, prioritizing prospective designs, external validation, and clinically interpretable models. We focus on tasks relevant to clinicians: lesion triage from clinical images, prediction of nodal metastasis on CT/MRI/PET, margin assessment with optical modalities, and histopathology-based diagnosis/grading. We also discuss implementation issues: dataset shift, bias, and reporting standards.

**Results:**

In clinical photographs, deep learning achieves high diagnostic accuracy for OSCC and oral potentially malignant disorders (OPMD) classification in single-center studies and shows promising generalization with multi-site external testing, yet performance still degrades on out-of-distribution images and under real-world artifacts. In radiology, radiomics and deep learning models improve risk stratification and prediction of cervical nodal metastasis beyond conventional imaging, particularly with multimodal feature fusion. Optical methods such as hyperspectral spatial frequency domain imaging and OCT combined with AI show feasibility for intraoperative margin assessment and in-clinic triage. Digital pathology models on whole-slide images approach expert-level classification for OSCC diagnosis and are beginning to predict malignant transformation risk in oral epithelial dysplasia; however, rigorous prospective validation remains scarce. Conclusion: AI systems for OSCC are maturing and clinically oriented. Before routine adoption, studies must demonstrate external validity, clinician-in-the-loop performance, calibration, and impact on time-to-diagnosis and patient outcomes. Pragmatic trials and transparent reporting are essential to move beyond proof-of-concept into equitable clinical benefit.

**Graphical Abstract:**

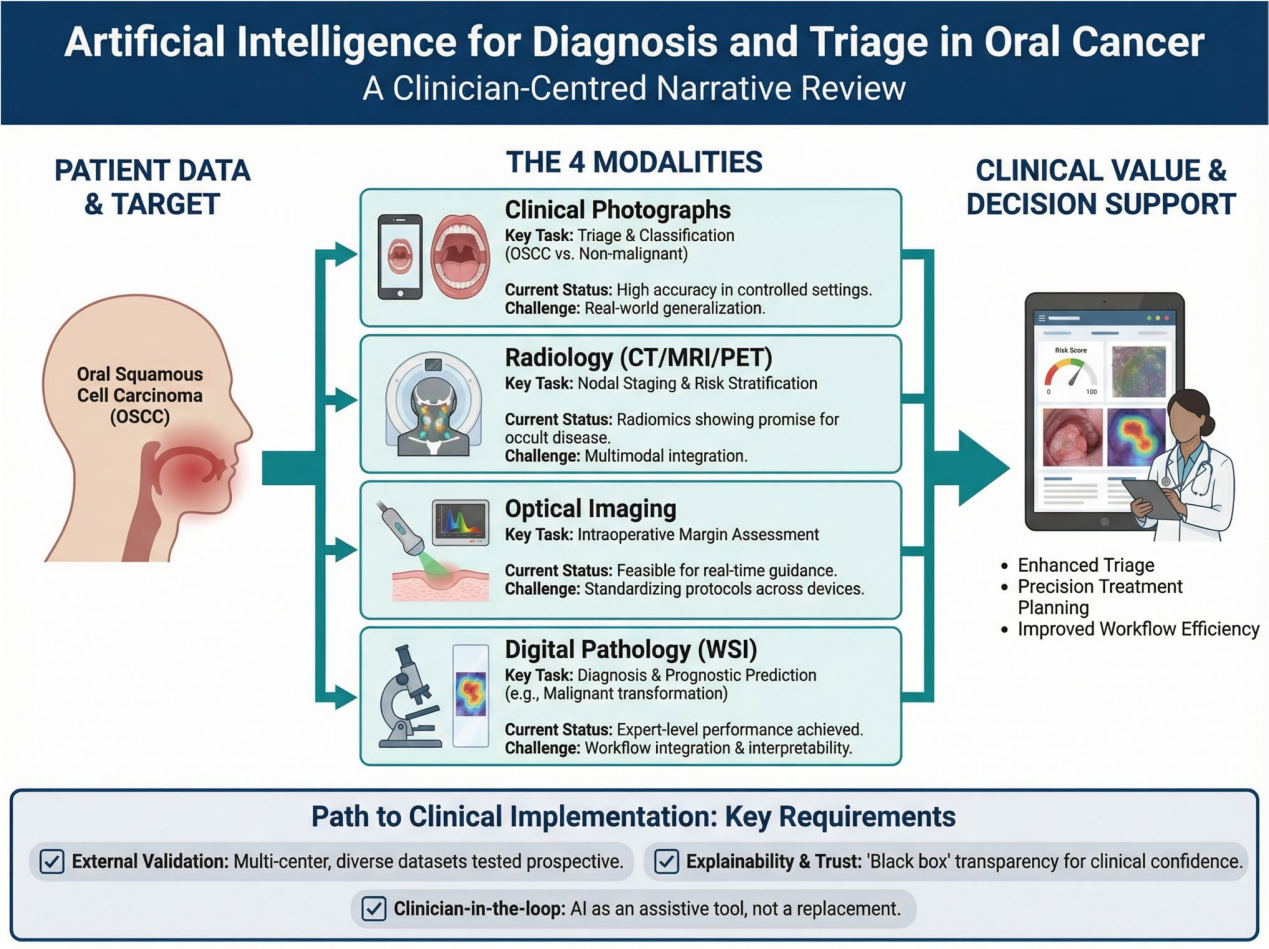

Overview of AI‑enabled diagnosis and triage in oral cancer across four modalities with validation priorities.

## Introduction

Oral squamous cell carcinoma (OSCC) accounts for the vast majority of oral malignancies and remains a major global health burden. Outcomes are tightly linked to stage at diagnosis; delays at the level of patient presentation and health-system processes contribute to advanced-stage detection in many settings. AI techniques—particularly deep learning—have transformed pattern recognition in imaging and are rapidly permeating clinical oncology. Foundational work in general AI-enabled medicine and image classification laid much of the groundwork for domain-specific applications in oral oncology [1–3]. For oral cancer care, the central question is no longer whether AI can classify images in a laboratory, but whether it can shorten the diagnostic pathway, improve risk stratification, and support decisions without amplifying inequities. Epidemiologic and diagnostic-delay contexts frame these challenges [4–8]. External validation is critical when translating artificial intelligence models into clinical practice. In this review, we define external validation as evaluation on data that are independent of model development and obtained from different institutions, time periods, acquisition settings, or patient populations. This matters because model performance often degrades under domain shift, and external validation is needed to support generalizability and transportability in real-world settings.

### Scope and approach

This is a narrative (not systematic) review of PubMed-indexed, peer-reviewed, English-language studies published through October 2025. We searched PubMed using combinations of keywords related to oral squamous cell carcinoma, oral potentially malignant disorders, artificial intelligence, deep learning, radiomics, computed tomography, magnetic resonance imaging, positron emission tomography, optical coherence tomography, optical imaging, and digital pathology.

We focused on four clinician-facing tasks because they map to common decision points in care and have a growing body of peer-reviewed evidence: (i) triage from clinical photographs, (ii) prediction of cervical nodal metastasis on CT/MRI/PET, (iii) margin assessment and chair-side triage with optical modalities, and (iv) histopathology-based diagnosis or grading using whole-slide images.

We included original studies with clearly defined reference standards and quantitative evaluation. We prioritized studies that reported external validation or multi-site cohorts and those that provided clinically interpretable performance reporting, including discrimination, calibration, and error analysis. We excluded preprints and non-peer-reviewed sources to minimize citation drift. Because this is a narrative review, we did not aim for exhaustive coverage; instead, we selected representative studies to illustrate evidence maturity and implementation constraints across modalities.

### Clinical photograph-based triage

A sustained line of work has explored AI classification from intraoral photographs captured by consumer-grade cameras. A landmark retrospective multicenter study showed that a convolutional neural network could identify oral cavity squamous cell carcinoma from clinical images with high accuracy and potential for telemedicine triage [9]. Subsequent reviews characterize common failure modes—lighting, blur, mucosal glare, and under-representation of darker mucosa—each of which degrades point-of-care performance [7, 10, 11]. Recent single- and multi-center datasets, including transformer-based approaches, reported improved generalization, and external testing for OSCC and oral potentially malignant disorders (OPMD) differentiation [9, 12, 13]. Complementing these image-centered models, a reader-style evaluation using an LLM (GPT-4) on clinical photographs—with and without clinical history—showed better performance when history was available, underscoring clinician-in-the-loop workflows [14]. In parallel, smaller pilot studies using object-detection frameworks demonstrate feasibility but need larger cohorts and external validation [15]. Methods tailored to early disease presentation continue to appear and may help address subtle lesions [16]. Meta-analytic evidence suggests that AI-assisted image assessment achieves high pooled accuracy across imaging tools, though heterogeneity and reference-standard differences remain [17, 18]. Representative clinical photograph studies and their validation characteristics are summarized in Table 1.

**Table 1:** Representative studies using clinical photographs for OSCC/OPMD classification and triage

First author (year); Journal	Country/centers	Design	N (images/patients; split)	Task	External validation	Primary performance (external test preferred)	Comparator
Fu (2020)[9]	China; 11 centers	Retrospective, multi-center	44,409 images; internal *n* = 401, external *n* = 402	Detect OCSCC from clinical photographs	Yes	External: AUC 0.935 (95% CI 0.910–0.957), Sens 89.6%, Spec 80.6%; internal: AUC 0.983 (0.973–0.991), Sens 94.9%, Spec 88.7%	7 oral-cancer specialists (comparable)
Kouketsu (2024)[12]	Japan; single center (outpatient clinic)	Retrospective image-based	1,043 images from 424 patients	Detect OSCC (and OSCC + leukoplakia) from clinical photos	No (not reported)	OSCC detection: Sens 93.9%, Spec 81.2%; OSCC + leukoplakia: Sens 83.7%, Spec 81.2%	—
Keser (2024); [15]	Türkiye; single center	Retrospective pilot (YOLOv5)	65 images; split 53/6/6 (train/val/test)	Detect oral cancer in clinical photos	No	Test: F1 0.667, Sens 0.667, precision 0.667	—
Schmidl (2025); [14]	Germany; single center	Prospective reader study (LLM assistant)	45 patients (histology proven)	AI assistant (GPT-4o) classifies OSCC vs non-OSCC from photos ± history	No	With history: OSCC Sens 100%, Spec 88.2%; leukoplakia Sens 93.3%, Spec 96.7%	Human readers (context-aware)

Reported metrics are as published in the source articles

*AUC* area under the ROC curve, *Sens* sensitivity, *Spec* specificity, *95% CI* 95% confidence interval, *OCSCC* oral cavity squamous cell carcinoma, *OSCC* oral squamous cell carcinoma, *OED* oral epithelial dysplasia, *OED MT* malignant transformation, *EV* external validation, *LLM* large language model

### Cross‑sectional radiology: nodal staging and risk stratification

**Fig. 1:**
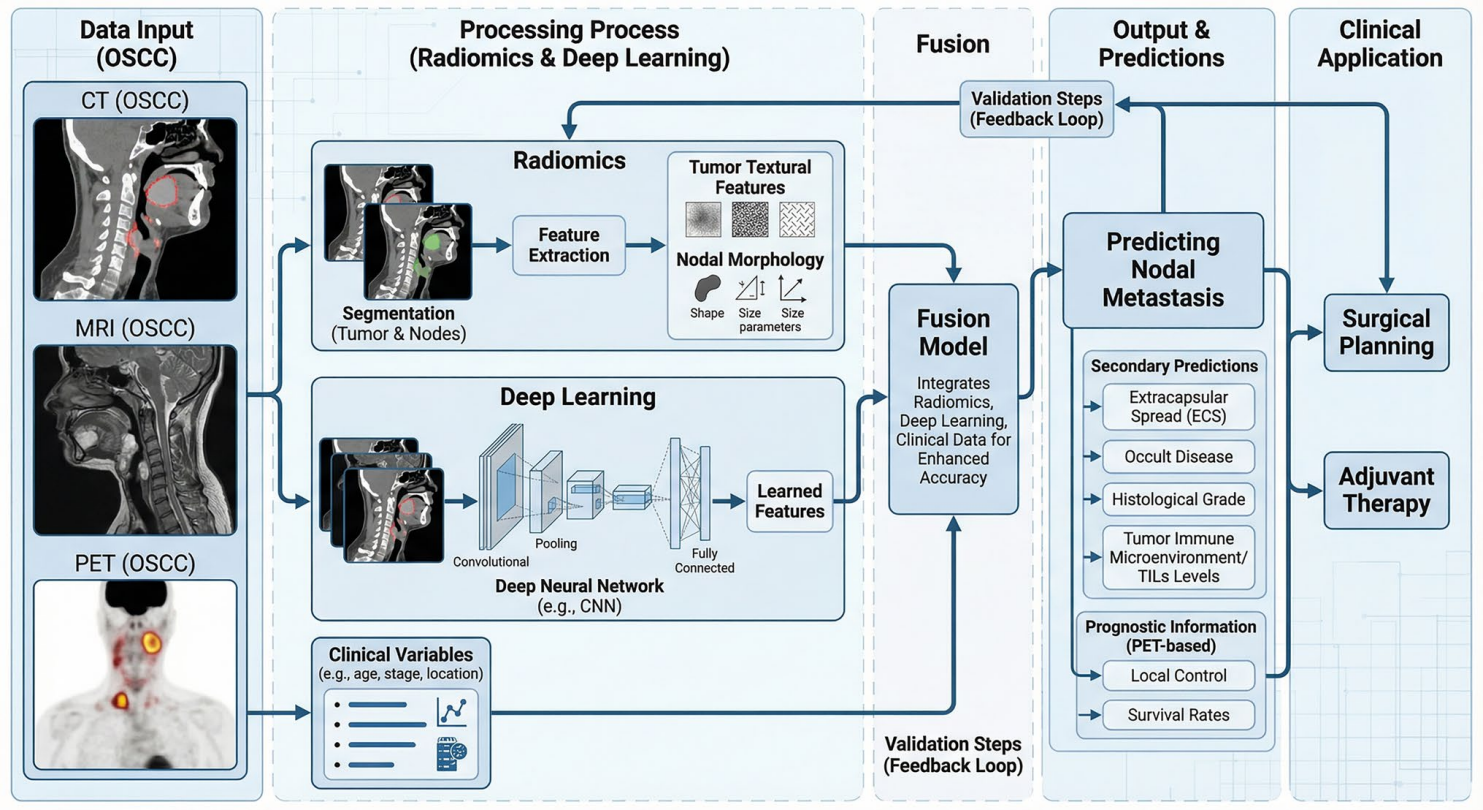
Radiology pipeline

In radiology, the main clinical aim is preoperative staging and risk stratification that informs surgical planning, neck management, and adjuvant therapy. Most oral cancer AI work, therefore, targets prediction of cervical nodal metastasis and related adverse features, and it secondarily supports prognostication and more standardized image interpretation. Figure 1 summarizes a typical radiology AI workflow, from image acquisition and feature extraction to model evaluation and validation.

Radiomics and deep learning applied to CT/MRI have repeatedly shown incremental value for predicting nodal metastasis, extracapsular spread, and occult nodal disease beyond radiologist assessment alone [19–21]. Models that integrate tumor textural features with nodal morphology and clinical variables tend to calibrate better than image-only approaches. PET-based radiomics adds prognostic information for local control and survival, and node-level classifiers can outperform semi-quantitative features [22, 23]. PET radiomics has also been used to predict histologic grade preoperatively in OSCC [24]. Beyond nodal staging and histologic grade prediction, MRI radiomics has also been explored as a surrogate of the tumor immune microenvironment; in a single-center pilot on oral tongue SCC, a machine learning model estimated tumor-infiltrating lymphocyte levels from preoperative MRI, suggesting feasibility but requiring external validation [25]. A recent meta-analysis indicates that hybrid PET/MRI performs at least comparably with PET/CT for primary and nodal evaluation, with overlapping confidence intervals in head-to-head analyses [26]. For broader clinical positioning of PET/CT in HNSCC, contemporary narrative reviews remain useful [27]. However, most reports remain retrospective and heterogeneous in endpoints and validation strategies, which limits transportability across sites and scanners. Table 2 summarizes representative studies and highlights gaps in external validation and clinically meaningful endpoints.

**Table 2:** Representative radiology studies applying radiomics/deep learning for nodal staging, prognostication, and PET-based risk assessment

First author (year); Journal	Modality/target	Design	Cohort (split/EV)	Primary metric (external test preferred)	Clinical endpoint
Lan (2024); [19]	MRI radiomics + DL; predict occult cervical LNM in early OSCC/OPSCC	Retrospective; multi-center with two external validation sets	*n* = 319; EV set1/2 AUCs reported	AUC train 0.928; test 0.878; external1 0.796; external2 0.834 (ResNet50 fusion)	Pre-op OCLNM prediction and prognosis
Bianconi (2024); [23]	FDG-PET radiomics; nodal status in HNC	Retrospective; per-node analysis	27 patients; 51 LNs; LOOCV	LR: PETBase AUC 0.840 vs PETRad AUC 0.880; Sens 68.0% → 72.0%, Spec 89.5% → 90.0%	Pathologic LN classification
Al-Ibraheem (2024) [26]; (meta-analysis)	FDG-PET/MRI (hybrid) for HNSCC	Systematic review and meta-analysis (12 studies)	638 patients overall	Pooled sensitivity/specificity: primary disease 93%/95% (per-patient); locoregional 93%/96%; nodal disease 89%/98% (per-lesion)	Diagnostic accuracy vs PET/CT or MRI
Martens (2020); [22]	FDG-PET radiomics; outcomes in LA-HNSCC	Retrospective; train/validation	Train *n* = 103; Val n = 71	Concordance index (validation): 0.645 (recurrence), 0.627 (metastasis)	Recurrence/distant metastasis/OS prediction
Nikkuni (2024); [24]	FDG-PET radiomics; histological grade of OSCC	Retrospective; supervised ML	Single center; retrospective *n* = 191	AUCs: LR 0.72, SVM 0.71, RF 0.84, NB 0.74, KNN 0.73	Grade prediction (pre-op)

*LNM* lymph node metastasis, *OCLNM* occult cervical lymph node metastasis, *HNSCC* head and neck squamous cell carcinoma, *AUC* area under ROC, *EV* external validation, *LR* logistic regression, *RF* random forest, *SVM* support vector machine

### Optical imaging for margin assessment and chair-side triage

Optical methods seek to bridge the gap between coarse white‑light inspection and definitive histology. Hyperspectral spatial frequency domain imaging (SFDI) analyzed with deep learning has been explored in preclinical and ex vivo settings to estimate subsurface margin thickness, suggesting feasibility for intraoperative margin assessment in the future [28]. Optical coherence tomography (OCT) meta-evidence and systematic reviews support high diagnostic accuracy for oral lesions, and AI-assisted OCT shows promise for chair-side triage when histology is unavailable [29]. These modalities are attractive precisely because they do not require large laboratories or complex post‑processing at the bedside—yet their impact depends on probe ergonomics in the oral cavity, training curves, and how confidently clinicians can act on model outputs while avoiding unnecessary biopsies.

### Digital pathology: from diagnosis to prediction

**Fig. 2:**
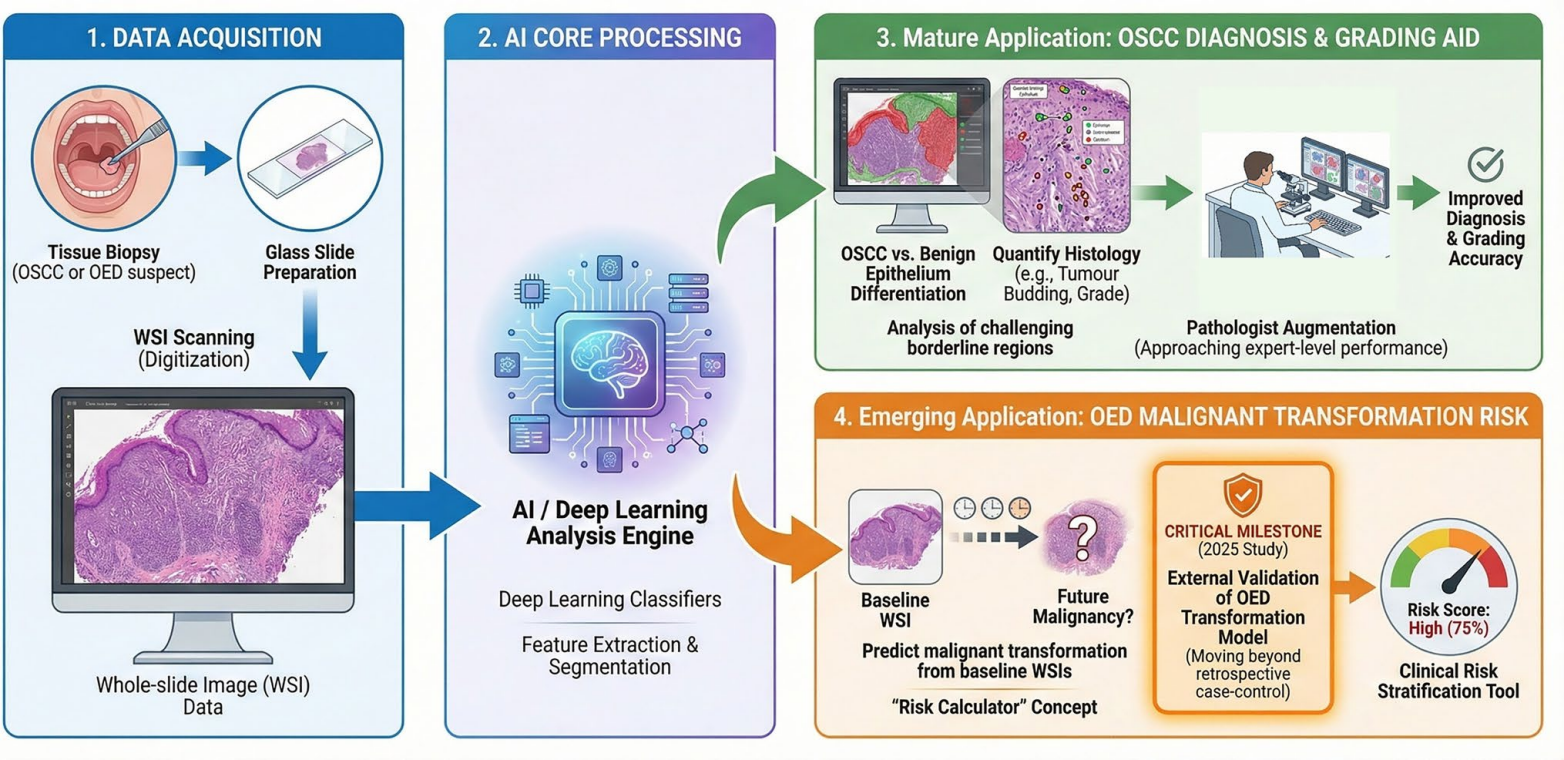
Digital pathology pipeline. End‑to‑end digital pathology workflow for OSCC diagnosis/grading and OED malignant transformation risk modeling

Whole‑slide image (WSI) analysis is the most mature AI application in OSCC. Figure 2 illustrates the end-to-end digital pathology workflow for OSCC diagnosis/grading and OED malignant transformation risk modeling. Multiple groups have reported approaching expert-level performance and augmenting pathologists in the histopathologic diagnosis of OSCC versus benign epithelium, and have begun to quantify tumor budding, grade, and other histologic predictors [30–32]. A study showed that deep learning classifiers can augment pathologists’ performance, particularly for challenging borderline regions [31]. Importantly, AI is starting to address prediction tasks that lock tightly to clinical decisions. Recent work in oral epithelial dysplasia (OED) proposes AI pipelines to predict malignant transformation from baseline WSIs—conceptually akin to risk calculators in other cancers. A 2025 study reported external validation for an OED transformation model, a critical milestone for moving beyond retrospective case–control classification [33]. Updates focused specifically on OED in digital pathology complement these advances [34]. Representative whole-slide image studies in OSCC and OED are summarized in Table 3

**Table 3:** Digital pathology studies

First author (year); Journal	Specimen/task	Design	Cohort	External validation	Primary metric	Clinical endpoint
Shephard (2024); [35]	WSI (OED); malignant transformation prediction (OMTscore)	Retrospective; internal CV + 2 external cohorts	Internal *n* = 193; external *n* = 89 (2 sites)	Yes (2 centers)	External AUROC 0.75 (recall 0.92); C-index 0.60 (*p* = 0.02)	Prognostic stratification for OED (MT risk)
Shephard (2025); [33]	WSI (OED); malignant transformation prediction (ODYN)	Development + external validation (3 centers; 3 scanners)	Train 358 OED + 105 controls; external 108 OED	Yes (3 centers)	AUROC 0.73 (external); C-index 0.63 (external)	Prognostic risk scoring for OED
Sukegawa (2024); [36]	Oral exfoliative cytology; CNN classification	Retrospective; repeated CV, probabilistic labels	14,535 patch images; 6 WSIs (LBC)	No	AUC up to 0.991; accuracy up to 0.988 across cases; prior baseline accuracy 0.902 (patch-level)	Screening-adjunct classification

Representative whole-slide image studies in OSCC and OED, including diagnostic classification and risk prediction

*MT risk* malignant transformation risk

### Integrative perspectives and clinician‑in‑the‑loop design

This section synthesizes evidence maturity and implementation considerations across modalities. To align the narrative with the evidence maturity map, we treat three study-design dimensions as the core indicators of clinical credibility: external validation, prospective evaluation, and clinically meaningful endpoints. In addition, we discuss cross-cutting factors that influence interpretability and reproducibility, including multimodal modeling, dataset diversity and equity, sample size, multi-center recruitment, transparency of preprocessing and model development, and appropriateness of evaluation metrics. Three patterns recur. First, external validation remains the exception; when present, performance typically drops relative to internal testing, as shown across clinical photographs, digital pathology, and radiology [9, 19, 33]. Second, multimodal approaches often improve discrimination and may yield more stable calibration than single-source models—mirroring how clinicians synthesize data across domains [19, 22]. Third, equity and generalizability must be designed from the start: datasets should reflect the full spectrum of oral mucosal pigmentation, dental restorations and prostheses, and the diversity of lesion mimics seen in general practice, not solely tertiary centers [7, 10]. Transparent reporting aligned with the TRIPOD (Transparent Reporting of a multivariable prediction model for Individual Prognosis Or Diagnosis) [37, 38] and STARD (Standards for Reporting Diagnostic Accuracy Studies) [39, 40] guidelines, including their AI-oriented extensions, and prospective impact evaluations anchored to outcomes, such as time-to-biopsy, stage shift, and avoidable re-excisions, are essential.

These patterns are summarized qualitatively in Table 4, which provides a directional snapshot of evidence maturity by modality and study design dimension. In brief, clinical photographs and digital pathology show emerging strength for external validation, whereas prospective designs and clinical impact endpoints remain scarce across all modalities; radiology (CT/MRI/PET) shows a similar profile, and optical imaging currently has sparse evidence on all three axes[17, 18, 29, 34].

**Table 4:** Evidence maturity matrix (qualitative)

Modality	External validation	Prospective design	Clinical impact endpoints
Clinical photos	○	–	–
Radiology (CT/MRI/PET)	○	–	–
Optical imaging	–	–	–
Digital pathology	○	–	–

Qualitative maturity of the evidence by modality and design dimension

Design dimensions: external validation, prospective design, clinical impact endpoints. Legend: ○ emerging, — sparse. Ratings reflect peer-reviewed literature up to October 2025 and are intended as a directional summary [9, 14, 17–20, 23, 27, 29, 34]

From an implementation standpoint, two challenges dominate: domain shift across devices, acquisition protocols, and clinical settings, and the need for trustworthy model behavior at the point of care. Domain shift arises from differences in camera type and lighting in clinical photographs, scanner and reconstruction settings in radiology, acquisition constraints in optical imaging, and staining and scanning pipelines in digital pathology. Mitigation requires standardized acquisition where feasible, robust augmentation, and site-aware external validation.

Explainability methods may support clinician acceptance, but post hoc attributions do not guarantee faithful explanations. Therefore, deployment should emphasize calibrated uncertainty, explicit failure-mode reporting, and monitoring for performance drift after rollout [41].

### Practical guidance for clinicians and services

For clinics exploring adoption, we recommend a staged approach. Begin with adjunctive triage applications for which the action is uncontroversial (e.g., flagging high-risk lesions for urgent assessment, standardizing photographic capture). Use shadow-mode audits to measure real-world false positive/negative profiles before allowing the system to influence decisions. For radiology services, prioritize models with open documentation, proven external datasets, and locked algorithms; integrate them into reporting templates that expose model uncertainty and rationales. For pathology departments, invest in robust WSI pipelines and metadata governance first; model selection comes second. Finally, define exit criteria—the thresholds at which an AI tool will be rolled back if drift, bias, or calibration failure emerges. Regulatory status should be considered explicitly. AI tools intended for diagnostic use may require medical-device regulatory authorization depending on jurisdiction. Within the studies included in this review, regulatory authorization and routine clinical deployment are not consistently described; this gap likely reflects limited multi-site validation, workflow integration constraints, and evidentiary requirements for authorization. We, therefore, distinguish research-grade prototypes from evidence that supports clinical deployment when summarizing each modality. Table 5 provides a concise checklist of minimum requirements and safeguards for clinical deployment.

**Table 5:** Minimum requirements and safeguards for clinical deployment of AI systems in oral oncology

Domain	Requirement/safeguard (minimum requirement)	Rationale/specifics
Phased rollout (staged approach)	Begin with adjunctive triage applications	e.g., Flagging high-risk lesions for urgent assessment or standardizing photographic capture
Pre-deployment validation	Use ‘shadow-mode’ audits before live deployment	Measure real-world false positive/negative profiles before the system is allowed to influence clinical decisions
Model vetting (radiology)	Prioritize models with open documentation, proven external datasets, and locked algorithms	Ensure transparency and validated performance
Model vetting (pathology)	Invest in robust WSI (whole-slide image) pipelines and metadata governance first	Model selection is secondary to the quality of the underlying digital infrastructure
Clinical integration	Integrate AI outputs into reporting templates that expose model uncertainty and rationales	Clinicians must be able to see the AI’s confidence and (if possible) its reasoning
Ongoing governance and monitoring	Define clear ‘exit criteria’ for rolling back the tool	Must continuously monitor for performance drift, bias, or calibration failure after deployment

### Limitations of the evidence

Most studies remain retrospective and single‑center; many lack pre‑registered protocols, clinical impact measures, or detailed error analyses. Performance is often reported as point estimates without decision-curve analyses or cost‑effectiveness. Publication bias and dataset reuse complicate pooled estimates. These limitations should temper enthusiasm and motivate stronger methodology rather than discourage exploration.

## Conclusion

AI for oral cancer diagnosis has progressed from promising prototypes to clinically meaningful tools across images acquired by clinicians, radiologists, and pathologists. The evidence base now supports adjunctive use for triage and risk stratification, with emerging signals for predictive modeling in OED. The next decisive step is not a marginal AUC gain but trustworthy, externally validated systems embedded in clinical workflows that shorten diagnostic delays and improve patient outcomes.

## Data Availability

Not applicable.
